# Beyond the diagnosis of drug-resistant Tuberculosis in Norway: patients’ experiences before, during and after treatment

**DOI:** 10.1186/s12889-024-19342-8

**Published:** 2024-07-06

**Authors:** Eline Storvig, Ingunn Harstad, Birgitta Ehrnström, Valentina C. Iversen

**Affiliations:** 1 Department of Infectious Diseases, Clinic of Medicine, St. Olavs Hospital, Trondheim University Hospital, Trondheim, Norway; 2 Department of Public Health and Nursing, Faculty of Medicine and Health Sciences, Norwegian University of Science and Technology (NTNU),, Trondheim, Norway; 3 Department of Pulmonary Medicine, St. Olavs Hospital, Trondheim University Hospital, Trondheim, Norway; 4 Mid-Norway Group for Sepsis Research, Clinic of Anesthesia and Intensive Care, St. Olavs Hospital, Trondheim University Hospital, Trondheim, Norway; 5 Department of Clinical and Molecular Medicine, Faculty of Medicine and Health Sciences, Norwegian University of Science and Technology (NTNU), Trondheim, Norway; 6 Department of Mental Health, Norwegian University of Science and Technology, Trondheim, Norway; 7 Nidelv Mental Health Center, St. Olavs University Hospital, Trondheim, Norway

**Keywords:** Drug-resistant tuberculosis, Psychosocial health, Patients’ experiences, Low-endemic countries, Qualitative research

## Abstract

**Background:**

This study aims to explore the varied experiences of patients with drug-resistant tuberculosis in Norway. The study emphasizes challenges and implications of being diagnosed with drug-resistant tuberculosis, including the impact on psychosocial health during the diagnosis, disease, treatment, isolation and recovery phases.

Norway is a low endemic country of tuberculosis. Most patients are immigrants, and some of them have recently arrived in the country. Patients undergoing treatment for drug-resistant tuberculosis endure prolonged and demanding treatment that could affect their psychosocial health.

**Methods:**

This qualitative study conducted 16 in-depth interviews with individuals aged 18 years and above who were diagnosed with drug-resistant tuberculosis. All participants completed the treatment between 2008 and 2020. Fourteen participants were immigrants, and eight of them had resided in Norway for less than four years before diagnosis. Data analysis followed the six-phase reflexive thematic analysis framework, focusing on identifying patterns in participants’ experiences, thoughts, expectations and attitudes.

**Results:**

The narratives of the participants highlighted the complexities of navigating the diagnosis of drug-resistant tuberculosis, treatment, side effects and life after treatment. Immigrants encountered additional challenges, including language barriers and adapting to new social environments. All participants reported experiencing physical health issues that additionally affected their mental health and social activity. Several participants had a delayed or prolonged diagnosis that complicated their disease trajectory. Participants with suspected or confirmed contagious pulmonary tuberculosis underwent hospital isolation for periods ranging from weeks to six months. The participants reported mental health issues, social isolation and stigma, however few were offered follow-up by a psychologist. Many participants had persistent problems at the time of the interviews. Three main themes emerged from the analysis: Delayed and prolonged diagnosis; Psychosocial impact of isolation during treatment; The life after tuberculosis.

**Conclusion:**

This study highlights the enduring impact of drug-resistant tuberculosis on patients and the significance of timely diagnosis, psychosocial support and post-treatment follow-up. The participants universally faced serious implications of the disease, including stigma and isolation. Participants who experienced delayed diagnosis, reflected on missed early intervention opportunities. We recommend further research in low endemic countries to evaluate the international and local recommendations on psychosocial support.

**Supplementary Information:**

The online version contains supplementary material available at 10.1186/s12889-024-19342-8.

## Introduction

Tuberculosis (TB) is an infectious disease caused by *Mycobacterium tuberculosis*, with pulmonary TB being the most common form and potentially contagious. However, TB can affect any organ in the body [1]. The World Health Organization (WHO) defines drug-resistant tuberculosis (DR-TB) as a TB disease caused by a *Mycobacterium tuberculosis* complex strain resistant to any of the TB medicines [2] Multidrug-resistant tuberculosis (MDR-TB) is defined as resistance to at least rifampicin and isoniazid [2]. MDR-TB has been associated with a prolonged and complicated treatment with severe side effects, and a prolonged hospital isolation when contagious [3]. Regimen for rifampicin susceptible and isoniazid resistant TB is usually not so complicated [2].

In 2022, WHO estimated 10.6 million new TB cases and 1.3 million deaths of TB. Additionally, WHO estimated 410,000 cases with rifampicin-resistant (RR-TB) strains [1].

In Norway with a population of 5.4 million, 174 people were diagnosed with TB in 2022, and among those, ten had MDR-TB. Immigrants were primarily affected, and 72 of them had stayed less than four years in Norway [4] The global TB situation and immigration significantly influence TB incidence and resistance patterns in Norway [4].

The WHO, Stop TB Partnership and International Union against Tuberculosis and Lung Diseases (The Union) highlight the need for psychosocial support and person-centred care for patients with DR-TB [5–7]. DR-TB has an impact on patients’ psychosocial health, and The Union defines psychosocial support as psychological, social and economic factors that strengthen people to get a diagnosis, to adhere to care plans and to complete a course of treatment [6] The Stop TB Partnership describes a people-centred approach that considers the needs, perspectives, and individual experiences of people affected by TB, while respecting their right to be informed and receive the best quality care based on individual needs [7, 8]. The Union published recommendations in 2021 to address the psychosocial needs of TB patients. The recommendations are person-centred, and recognize the importance of holistic care aiming to create a supportive environment for patients. The target groups for the recommendations are healthcare personnel, governments and TB support providers [6].These approaches prioritize the individuals’ unique needs and fosters an effective and compassionate healthcare environment [6, 7].

A systematic review and meta-analysis from 2018 found high prevalence of mental disorders and common social stressors like stigma, self-isolation or lack of social support among DR-TB patients. This highlighted the necessity to integrate assessment of mental health, social protection and social support in TB guidelines and in clinical TB-treatment [9]. Other researchers reported that TB treatment can have long-term adverse effects on patients’ health, even years after successful treatment [10–12]. This underscores the need for continuous support and monitoring survivors of TB to address these persistent challenges [10, 12].

Assessing patients’ psychosocial health includes examination of stigma associated with tuberculosis (TB stigma). TB stigma contributes to feelings of isolation, shame and fear of discrimination. A review in 2022 concluded that there is limited evidence on interventions aimed at reducing TB stigma [13]. The Stop TB Partnership has developed a framework to understand and assess different types of stigma [14]. Self-stigma encompasses when TB patients accept negative stereotypes and behave in a manner that endorses them. Enacted or experienced stigma reflects the range of stigmatizing behaviour directly experienced by individuals with TB or actions by others, such as discrimination, rejection, or isolation, in various settings. Anticipated stigma (or perceived stigma) refers to the concern that one will be devalued following a TB diagnosis. A person with TB may fear that this stigma could be severe enough to impact their access to TB care [14, 15].

This study aims to understand the diverse experiences of patients diagnosed with resistant tuberculosis in Norway, particularly among immigrants. The study emphasizes challenges and implications of being diagnosed with drug resistant tuberculosis, including the impact on psychosocial health during the disease, treatment, isolation and recovery phases.

## Method

### Study design and data collection

The qualitative research approach employed semi-structured interviews to delve into the experiences of patients. Thematic analysis was used to examine the data, focusing on identifying patterns in participants’ experiences, thoughts, expectations and attitudes. This method was chosen to understand how participants perceived their illness, interacted with the healthcare system and managed the life throughout their diagnosis, treatment and recovery phases [16, 17].

The study included individuals above 18 years who had been diagnosed with DR-TB. Participants were sourced from TB registers at three university hospitals in Norway, covering the period from 2008 to 2020. All registered patients to whom the study gained access, had completed the DR-TB treatment. Out of 31 individuals invited, 16 participated, comprising five men and 11 women aged between 19 to 71 years, with a mean age of 33.6 years. Fourteen participants were immigrants. Table 1 presents the overview of the participants.

**Table 1:** Overview of the participants

	MDR TB (%) (*n* = 8)	DR-TB (%) except MDR-TB (*n* = 8)	Total (%) (*n* = 16)
**Gender**			
Male	2 (25)	3 (37.5)	5 (31.25)
Female	6 (75)	5 (62.5)	11 (68.75)
**Age group (years) during treatment (mean 34 years)**			
20–39	8 (100)	5 (62.5)	13 (81.25)
40–59		2 (25)	2 (12.5)
> 60		1 (12.5)	1 (6.25)
**Civil status and children < 18 years during treatment**			
Single	3 (37.5)	3 (37.5)	6 (37.5)
Married/ partnership	5 (62.5)	5 (62.5)	10 (62.5)
Children < 18 years	5 (62.5)	3 (37.5)	8 (50)
**Origin**			
Africa	5 (62.5)	3 (37.5)	8 (50)
Asia	1 (12.5)	2 (25)	3 (18.75)
Europa	2 (25)	3 (37.5)	5 (31.25)
**Education (years)**			
No school/ < 6		1 (12.5)	1 (6.25)
6–10		1 (12.5)	1 (6.25)
11–13	6 (75)	1 (12.5)	7 (43.75)
> 13	2 (25)	5 (62.5)	7 (43.75)
**Years in Norway before treatment for immigrants (** ***n*** ** = 14)**			
< 1 year	2 (25)		2 (14.3)
1–4	3 (37.5)	3 (50)	6 (42.9)
5–9	2 (25)	1 (16,7)	3 (21.4)
> 10	1 (12.5)	2 (33,3)	3 (21.4)

Interviews were conducted in two phases: ten in the autumn of 2019 and six in the autumn of 2021. Participants were provided with written details about the study’s purpose, procedures, their rights and ethical considerations, in Norwegian or English, with adjustments made for language barriers. Interviews, conducted both in-person and via video call, explored their TB experiences and psychosocial health impacts. The interviewer was the first author (ES). All interviews were audio recorded and transcribed, and durations were ranging from 42 to 88 min.

### Settings

The Norwegian TB control program provides a comprehensive health service guide for diagnosing and treating DR-TB. All individuals arriving in Norway from high-incidence countries of TB, who plan to stay in Norway for more than three months, and all asylum-seekers independent of length of stay, are screened for tuberculosis with chest radiograph and/or Interferon-γ release assay (IGRA) [3, 18]. If the initial screening indicates a possible TB-infection, the individual is referred to the specialist health service. Additionally, individuals may be referred based on TB-suspected symptoms or in the process of contact tracing of those in close contact with a newly diagnosed TB patient [3]. In the case of a confirmed infection with DR-TB, treatment is based on the guidelines and resistance pattern. For many patients with active TB-infection, a period of hospitalization is needed due to the seriousness of the infection and to ensure that the treatment is effective and tolerated. The length of hospitalization varies greatly. Those with contagious, pulmonary TB are kept in hospital isolation for several weeks. The patients who are both contagious and infected with drug resistant TB often need more than a month in isolation. Once patients are considered non-contagious, they are discharged and continue treatment on an out-patient basis [3, 18]. Diagnosis and treatment for TB are free of charge regardless of residence permit status [18].

### Analysis of data

Analysis was conducted in accordance with six detailed phases in reflexive thematic analysis, described by Braun and Clarke [16]. 1) ES read the transcripts to become familiar with the data, and then re-read them to identify units of the text referring to challenges encountered by participants before, during and after being diagnosed with DR-TB. 2) Case by case ES coded units into analytic categories by using the software qualitative data analysis system, NVivo [19]. NVivo facilitated the structured coding of the interview transcript by labelling and creating categories for sections of data in the dataset [19]. ES and the last author (VI) then reviewed each category for coherence and data adequacy. 3) By comparing categories across the cases and referring to the full interviews, ES grouped the categories into initial themes by merging sub-categories. 4) As part of further development of themes, ES presented preliminary themes to all the authors, who reviewed and gave input on the themes. 5) ES and VI functioned as auditor to refining and defining the themes before 6) final writing up. The resulting themes were then reviewed again and discussed in the member group. A study participant with English as native language, considered the results before final writing up.

### Ethics

The ethical approval for the study was granted by Regional Committee for Medical and Health Research Ethics (REK) Central Norway (REK2018/1813). The approval from REK applies to the three included hospitals. A privacy impact assessment (DPIA) was carried out at St. Olavs Hospital, Trondheim University Hospital. Participation was voluntary, and written informed consent was obtained. The participants were informed that confidentiality and anonymity were safeguarded. The information and consent form were prepared according to guidelines of REK.

### Reflexivity

The first author is tuberculosis coordinator with 10 years of experience in the TB field. The research group has extensive clinical and research backgrounds with both TB patients and immigrants, which shaped the study’s design and analysis. These experiences contributed to a deep understanding of the subject matter and facilitated a nuanced approach to exploring the participants’ experiences with DR-TB. Measures to raise awareness of distinctive features in the researchers’ interpretations included writing memos and notes, as well as supervision and member checking meetings. The authors emphasized reflexivity in recognizing their own influence on the research. This reflective practice enriched the study’s insights, grounding its conclusions in both empirical evidence and informed empathy [16].

## Results

The participants’ narratives highlighted the challenges of navigating the TB diagnosis, treatment, side effects and life after treatment. The participants were affected by DR-TB in different organs, and they experienced a wide range of treatment durations. Table 2 presents an overview of patient and disease characteristics.

**Table 2:** Overview of patient and disease characteristics

	MDR TB (%) (*n* = 8)	DR-TB (%) except MDR-TB (*n* =)	Total (% of total) (*n* = 16)
**Start of treatment (year)**			
2009–2014	4 (50)	2 (25)	6 (37.5)
2015–2020	4 (50)	6 (75)	10 (62.5)
**Duration of treatment (months)**			
9–12	3 (37.5)	6 (75)	9 (56.3)
13–18	1 (12.5)	2 (25)	3 (18.7)
> 19	4 (50)		4 (25)
**Site of infection**			
Lung	6 (75)	2 (25)	8 (50)
Abdomen	1 (12.5)	2 (25)	3 (18.75)
Skeleton/ skin		2 (25)	2 (12.5)
Lymph node	1 (12.5)	2 (25)	3 (18.75)
**Participant reported healthcare delay**			
Participants	3 (37.5)	5 (62.5)	8 (50)

**Fig. 1:**
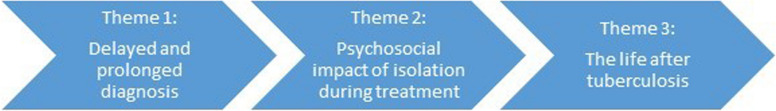
Main themes

Immigrants faced additional challenges, including language barriers and adapting to new social environments. All participants reported one or more physical problems like dizziness, nausea, hearing loss, nephropathies and other serious conditions. Physical problems further affected their mental health and social activity. MDR-treatment led to increased occurrence of side effects. Many participants reported persistent physical and mental problems many years after completed treatment. The thematic analysis revealed three main themes (Fig. 1), showcasing the diverse experiences and challenges faced by DR-TB patients.

### Theme 1: Delayed and prolonged diagnosis

The first theme highlighted the participants’ struggle in the start phase of diagnosis with delayed and prolonged diagnosis. By delayed diagnosis, we mean a situation where a General Practitioner took longer than appropriate to refer the patient to a hospital or specialist for further diagnostic testing. By prolonged diagnosis, we mean a situation where the patient had to wait for DR-TB treatment due to misdiagnosis or waiting for test results. Two participants were referred for further diagnostic testing after arrival screening. Eight participants reported delayed diagnosis and encountered this challenge despite experiencing severe pain, serious symptoms of lung disease or abdominal issues. Several participants reported prolonged diagnosis in the specialist healthcare. Some participants received cancer as a tentative diagnosis, and other participants had to await resistance pattern analysis before commencing treatment. These delayed and prolonged diagnostic journeys were described as a very exhausting period by the participants.

#### Delayed diagnosis despite seeking healthcare

Despite proactive efforts to seek medical help from the primary healthcare, eight participants encountered problems in securing hospital referrals and did not receive timely treatment. These participants reported severe pain, serious symptoms of lung disease or serious abdominal issues. One participant evaluated diagnosis, treatment and the isolation period like this:

If I want to summarize, general practitioner problem. Treatment went perfectly well for me. I have no complaints, because I knew what was going on. If it is unknown, there is a problem. [Participant 9 – Man, p.11]

Three participants described persistent coughing, fever, weight loss up to ten kilos and experienced that their General Practitioner did not take their symptoms seriously.

So, I came again and told her, I am very sick, I am coughing a lot, I have a fever and stuff like that. So, she said, maybe you have a cold ... take cough syrup or something ... she did not help anything, she did not know ... I could not go to school or do anything. [Participant 16 – Woman, p.29]

Another participant, with TB in the cervical vertebrae which entailed pain, stiffness and minimal muscle function in one arm, contacted her General Practitioner many times asking for referral to hospital. She reported that she needed to pretend losing consciousness to secure admission to the hospital for further medical diagnostic.

I could not sleep, did not eat, and I just sat at night … it was just a pain … doctor said: I do not see anything. So, in the end my husband called an ambulance ... he said, she is unconscious. That is why I came to hospital … I went to him (General Practitioner) until I got a free pass … If better knowledge had been given for him. [Participant 10 – Woman, p.15]

Participants with abdominal TB found it challenging to pinpoint when the TB symptoms began, as they had experienced stomach issues many years before receiving a diagnosis. One of them experienced that her General Practitioner dismissed her abdominal problems as solely psychological.

I got worse and worse. I could not eat food ... she did not listen to me. So, then she ended up throwing me out of the doctor’s office, because I thought she did a bad job ... I have never known that you can have TB elsewhere than in the lungs. And I don’t think very many doctors know that. [Participant 15 – Woman, p.2]

Two participants with delayed diagnosis described persistent cavities in their lungs after completed treatment. Several participants expressed the belief that earlier intervention by their General Practitioners might have prevented serious illness and mitigated the extensive sequelae of their DR-TB.

I should have been diagnosed much earlier because then I might have not gotten it in my back and been so bothered. [Participant 12 – Woman, p.7]

#### Prolonged diagnostic period

Some participants associated their TB with cancer and death, and two participants reported receiving a tentative cancer diagnosis. One participant, a mother of two small children, shared her feelings of powerlessness when healthcare personnel informed her about a potential cancer diagnosis. She said that she and her spouse wondered what a cancer diagnosis would bring, as they were new in Norway and had no other family nearby. When later receiving information about TB instead of cancer, she felt more optimistic that she would manage the treatment well. Another participant reported that healthcare personnel consistently concluded that it looked like cancer, and she obtained much information about a cancer diagnosis with high mortality.

They always concluded that it looked like cancer … I was mentally quite out of it, because it was a disease, I read up and down about, when I received the cancer diagnosis. And it was a very little survival. [Participant 15 – Woman, p.3]

Some participants were aware that they had TB but awaited resistance pattern analysis. Two of them were contagious, waited one month in hospital isolation before starting treatment and experienced severe disease development.

That time was very difficult for me. I had to wait a month for the medicine. A month, they said, no, that medicine have no effect, we are going to start another medicine. [Participant 16 – Woman, p.2]

### Theme 2: Psychosocial impact of isolation during treatment

Eight participants experienced long lasting hospital isolation, and most of the participants experienced social isolation. Social isolation stemmed from various factors, including the disease itself and hospital isolation. The participants described that isolation had a significant effect on their psychosocial health.

#### Hospital isolation

Hospital isolation imposed an additional psychosocial impact on the participants. All isolated participants had knowledge about the necessity of this approach and mentioned important justifications, such as protecting children, families, communities and healthcare personnel. Several participants emphasized that receiving regular updates from a trusted physician was of great importance to manage this period. One participant expressed a desire for more frequent information and stressed the specialists’ responsibility to communicate their knowledge.

I think this is the work that any doctor in hospital always should do, when they got something ... to inform the patient … He should focus on his seriousness, not only dictation, work and other stuff, because at that time they did not give me a clear answer. [Participant 3 – Man, p. 5]

Some participants experienced hospital isolation far from their homes and seldom received visits from their families. One of them wanted to avoid these meetings and painful farewells with her young children. However, healthcare personnel supported her and said that she and her children needed these meetings.

My daughter was two years old. She wanted to stay with me, she wanted to kiss me ... So that time was very hard for me … I told the doctors, I don’t want them to come ... However, no, they said, they need you. [Participant 16 – Woman, p. 11]

Some participants appreciated that healthcare personnel recognized their sadness and spent time with them for conversation and social recreation. Everyone emphasized the importance of being able to leave their isolation rooms, spend time outdoors and meet their family and friends. However, some participants mentioned not being permitted to go outdoors for the first few weeks or even up to two months. Other participants reported no restrictions of getting outdoor and had access to a park directly from their isolation rooms. These participants also had the opportunity to meet other TB patients and peers in this park. One participant who had recently arrived in Norway, stated that access to fresh air protected her against depression.

After three weeks, they said I could go out. I was looking forward going out ... I was very, very sad, every day, so I had to think of something that made me happy … If I did not, I would get crazy. [Participant 4 – Woman, p. 8 and p. 10]

Several participants highlighted the importance of internet access and staying connected with the outside world.

#### Social isolation

Several participants reported social isolation even after being discharged from the hospital. Contributing factors included the disease itself, side effects of the medication, mental health issues, stigma, a lack of awareness within the community, and their own fear of being contagious. Side effects as nausea and dizziness kept some participants in bed many hours after taking medications. One participant spoke about some sort of a traumatic experience due to nausea which contributed to social changes like stopping university education for one year and having minimal contact with friends.

Four participants announced that they had depression during the treatment, but only two were offered follow-up by psychologist. One participant reported social isolation during treatment and linked this to mental problems and also post-traumatic stress disorders after wars. He neither mentioned that his psychosocial needs were mapped, nor receiving any psychological follow-up during treatment. One participant, who described social isolation, needed to ask several times to meet a psychologist. She felt paranoia, and it was important for her that a psychologist told her, that some of the TB-medicine could affect her mental health.

I asked several times to see a psychologist, and finally I got to talk to a psychologist, who I did not think really understood me. I remember getting paranoia ... Nobody had told me about these side effects. [Participant 15 – Woman, p. 6 and p. 9]

Some participants avoided social contact due to their previous experiences of stigma, like people keeping distance.

They refused to eat at the same table with me ... I tried ... to meet others, so I just, keep it myself, I don’t go out anymore, after that. [Participant 4 – Woman, p. 8]

Several participants did not expect others to understand their complicated situation and found it challenging to explain about their DR-TB.

You become very isolated. I wasn’t social, I did not talk to people … You do not encounter understanding and people do not understand what you’re going through, so it just becomes tiring to deal with people. [Participant 15 – Woman, p. 9]

One participant said that she chose not to disclose her disease because she wanted to avoid appearing weak. Another participant had experienced racism in her native country and wanted to protect herself against racism in Norway.

I have experienced racism in (her native country) ... When I came to Norway, I also thought that it is usually immigrants, who are infected like that. What do they think about us coming with illness? [Participant 14 – Woman, p. 10]

One participant with non-contagious abdominal TB opted not to tell anyone to prevent worrying his schoolmates. He had a tough time and did not manage to follow his study program during the disease. Two participants reported encountering stigma while visiting outpatient clinics and perceived that healthcare personnel lacked knowledge about TB.

The gynaecologist was not too happy that I came ... I realized that. I was angry … the doctor, acted as if he had no good knowledge … I felt badly treated. [Participant 13 – Woman, p. 9]

One participant who returned home to his wife and two young children after four and a half months of hospital isolation, described the fear of still being contagious.

There were a lot of children, and if the bacteria remain, I thought things like that … You know it when you’re cured, however, you’re not cured, but infection-free, but anyway it’s a bit like that. [Participant 6 – Man, p. 11]

Several participants chose not to inform their families about their illness to spare them worries, despite the disease seriously affecting them.

The doctors, they asked me to tell them (the family in his native country), so they could go to diagnosis. … I did not want to do that … If I told them, especially my mum … another sickness so ... but if I told them when I was in hospital, it would be hard for them.” [Participant 3 – Man, p. 6]

Several participants experienced that openness highlighted their needs, protected them against social isolation and supported them through the TB-period. One participant did not feel any shame, because TB is airborne, and it was nothing she had done. Some told that the contact tracing in the municipality, made their contagious TB disease visible for their network. No participants complained about this procedure.

### Theme 3: The life after tuberculosis

All participants reported that they had completed treatment, and nobody had experienced relapse after being cured in Norway. The participants described physical- and psychosocial impacts and a fear of relapsing.

#### Changes in life after treatment

All participants mentioned that disease and treatment contributed to persistent problems and changes that were attached to psychosocial impacts, pain, weakness and deafness. Some participants described it like never regaining strength and nothing feels as before.

In the left arm and leg ... cannot lift with it ... the foot, still hurts a little, but these are worse sometimes and get better sometimes. Nothing feels as before, yeah. [Participant 1 – Woman, p. 12]

One participant paused the school for four years due to pulmonary sequelae and breathing problems. Several participants said that it was troublesome to explain the lost years, and why they did not manage the normal educational- and job progresses. Some participants still worried about being contagious many years after completing treatment, and a grandfather talked about avoiding close contact with grandchildren. A father told that when his children had a cold, he still thought about his past MDR-TB contagious period.

If the children have a cold and such, and then you start thinking … this feeling will be in my whole life. [Participant 6 – Man, p. 13]

Similarly, another participant remained preoccupied with her contagious period, which had led to a child becoming infected. Despite undergoing mandatory TB screening and receiving no restrictions working with children, she still felt guilty.

It is still uncomfortable, that I know that children is infected. [Participant 13 – Woman, p. 3].

One participant experienced TB relapse ten years after being cured in his native country and thought that this could occur again.

Why did it come back after all these ten years? Now, still I am afraid, if it could come back after some years, or is it already destroyed in my body? [Participant 3 – Man, p. 7]

Two other participants said that they felt healthy shortly after stopping the medications and continuing their jobs and other activities.

#### Carrying out the burden of the disease

A participant described losing years of her life, when she was newly arrived in Norway and wanted to focus on family, education, job and friends.

Sometimes still I cry, because I feel like I lost some years of my life ... If I did not get the illness, perhaps I could study Norwegian earlier, perhaps I could have gotten a job earlier, perhaps I could have a network earlier ... So yes, it affected me, and my life. However, I could not turn back to the, I already have missed. We must live, what is given for us …, it scared me for life… if I cannot accept it, it will be my burden, so I just try to move on. [Participant 4 – Woman, p.16]

One participant reflected on how TB made it clear to him that life could be gone very quickly, prompting a change in his perspective, and he started to take everything from life. Another participant had learned that she could follow her goal to complete her education at the university despite deafness, as side effects of MDR-TB medications, and other complicated events in her life. She experienced problems accepting acquired deafness and still wondered why they had given her medicines, which contributed to this outcome.

I wonder why I got injuries for the rest of my life in a country that is so modern. I wonder about that all the time … I wonder how my life could be now ... and the years when I was ill, meant that I could not learn anything, it is almost three years. I think I could have finished university by now ... I am glad that I am healthy. I am also sad that I experienced that. [Participant 8 – Woman, p. 2 and p. 19]

One participant said that she did not focus on the lost years and wanted to look forward. The most important thing was to cure the TB, follow physicians’ advice and be there for her family and children. Another participant described that she was struggling for several years to return to her job and normal life.

It makes me a little sad that they do not take the patients seriously, just listen to them a little more. And not just wrapping everything up, that it’s just mental. [Participant 15 – Woman, p. 8]

This participant compared TB and cancer, and said if she had had cancer, there would have been many available support options. She experienced that when she had completed treatment for MDR-TB, healthcare personnel informed her that she was healthy, and that everything was fine. She claimed that it was difficult to receive understanding and support to manage life after completing TB treatment.

Who guides you through the build-up period? … With cancer and other types that are quite well known, you get a lot of offers, don’t you, and understanding. However, when it comes to TB, they just think you are healthy. That everything is good. [Participant 15 – Woman, p. 7 and p. 9]

## Discussion

This study aimed to delve into the experiences of patients with DR-TB in Norway, shedding light on the psychosocial impacts they encountered. Using a qualitative approach, the research facilitated a deep exploration of the nuanced and varied experiences perceived by the participants. The study revealed that DR-TB significantly impacted individuals across different backgrounds, affecting both immigrants and Norwegian-born individuals. The severity of the disease, side effects of medicines, isolation and stigma contributed to the hardships faced by patients. Notably, eight participants experienced delayed diagnosis which negatively affected them during and after treatment.

### Healthcare delay

Norway’s provision of free TB screening, treatment, control, and good access to TB diagnostics [18], is a significant measure to support timely diagnosis and treatment. However, many participants, whether dealing with pulmonary or extrapulmonary DR-TB, experienced that primary healthcare personnel did not recognize their serious TB symptoms, resulting in healthcare delay. Some participants wondered how the delayed diagnosis impacted the disease process. In this study, only two participants got the diagnosis through arrival screening, and this underscores the crucial need for primary healthcare providers in low-endemic countries, such as General Practitioners, to accurately recognize TB symptoms and not misinterpret or underestimate the severity of the conditions. A study from Norway in 2018 highlighted that General Practitioners seldom had consultations with TB patients and indicated a gap in TB-specific knowledge within primary healthcare. This gap underscores the need for enhanced training and resources to better manage the TB diagnosis in the cascade of care [20]. Early recognition and addressing of symptoms is a cornerstone of the WHO’s strategy to combat TB, emphasizing the critical need for heightened awareness and prompt action in the healthcare to improve outcomes [21].

Healthcare personnel need to consider the unique challenges faced by immigrants with serious illnesses to fully understand and address their challenges. Some previous publications offer valuable insights and recommendations and emphasize the importance of culturally sensitive care, accessibility, awareness and the need to effectively support immigrants dealing with TB [22–24]. A study from Portugal (2021) regarding delayed diagnosis of active pulmonary TB observed that patients’ delay, defined as the postponement of seeking healthcare by patients, was more prevalent than healthcare delay. Additionally, immigrants were more likely to experience delay in accessing healthcare [25]. In our study both immigrant participants and one Norwegian participant reported healthcare delay. Although they did not specifically mention patient delay, those with abdominal TB reported that their abdominal symptoms had persisted for several years. Our study identified that healthcare delay was frequently attributed to a lack of awareness and knowledge among General Practitioner in primary healthcare. Although the precise factors leading to this delay remain unclear, this study identified insufficient referral to specialist healthcare as a significant contributing factor.

### Psychosocial impact

The participants described facing a challenging period during diagnosis, treatment, isolation and recovery, with four participants reporting feelings of depression. Our findings on depression align with global data indicating a high prevalence of comorbid depression among TB patients [9, 26]. A publication of Sweetland from 2017 highlighted that clinical depression in TB patients can be triggered by the disease itself, associated stigma and side effects from TB medications. Additionally, the publication noted that TB can exacerbate existing social vulnerabilities, emphasizing the complex interplay between the disease and the broader socioeconomic factors affecting patients [26]. In our study fourteen of the participants were immigrants and belonged to a minority. Healthcare workers need to consider the complexity that might arise when minorities are affected with TB, and include both existing vulnerabilities and socioeconomic factors in the mapping. Psychosocial approaches, such as monitoring and awareness of patients’ psychosocial health, were integrated into the Norwegian TB guidelines in 2023, signifying a recognition of the importance of addressing these aspects of TB care [3].

Tuberculosis is associated with significant stigma, which remains a major challenge in TB control. Addressing TB stigma is crucial for improving patient outcomes and the effectiveness of public health strategies [13, 14]. Participants in our study reported stigma both within their social network and in hospital settings. No one mentioned concerns about stigma affecting access to TB healthcare. In addition, some participants felt unease knowing that they were contagious, contributing to feelings of shame or guilt. The participants did not report any targeted healthcare measures, such as public information, to protect against stigma. It is essential for healthcare personnel and collaborators to support patients in transforming self-stigma to self-worth. A systematic mapping review conducted in 2016 found limited studies on TB stigma in low-incidence countries, compared to the extensive evidence in the HIV field [15]. This indicates a need for more studies on TB stigma as a psychosocial determinant of health to better understand its impact and to develop effective interventions.

The long periods of hospital isolation experienced by participants raise concerns about their psychosocial health. With the introduction of new and improved medicines, the necessity and duration of such isolation should be re-evaluated [2]. The WHO’s Ethics Guidance for the Implementation of the End TB Strategy advises against isolating TB patients without first exploring all options to ensure treatment adherence and only under specific conditions [21]. Participants valued measures that improved their management during isolation, including access to fresh air and visits from family. Establishing a trust-based relationship through effective interaction and clear communication is crucial for patient support and management during the TB treatment process [5]. The experiences of getting outdoors from isolation varied among participants, with direct outdoor access from isolation rooms being a significant factor in their health. Healthcare personnel must acknowledge and address the psychosocial needs of TB patients during isolation and upon discharge, ensuring clear communication about their situation and the care plan to mitigate further psychosocial difficulties.

The WHO’s recommended all-oral short regime for MDR-TB from 2022 has great significance for patients with MDR-TB [2, 27]. A publication of Furin et al. from 2019 noted that although new tools and treatment regimens have been introduced, advances must also be made in providing high quality, person-centred care to protect against morbidity and stigma associated with tuberculosis [28]. Patients may still experience side effects from medications, need isolation when they are contagious and associate TB with a serious disease and stigma, despite introduction of new medicines and a shorter regime. Patients with MDR-TB treated prior to the introduction of all-oral short regime carry their own experiences and consequences, highlighting the ongoing need for supportive care and attention to individual experiences.

### Challenges after completed treatment

Several participants struggled to regain their physical and psychosocial health after completion of DR-TB treatment. One participant reported a lack of rehabilitation support after completed treatment and compared the TB care with the cancer care. The ‘Cancer association’ in Norway offers a variety of activities to cancer patients throughout the country [29]. A systematic review from 2021 quantified the prevalence and types of permanent TB related disabilities and reported that the most common disability was mental disorders [30]. A study from 2020 evaluated permanent respiratory TB sequelae in Mexico and Italy and pointed to the necessity of pulmonary rehabilitation [31]. Long-term health impacts emphasize the importance of monitoring comorbidities such as permanent disabilities and mental disorders, underscore a broader need for comprehensive post TB care and ensure a holistic approach to patient recovery and health [30–32]. Healthcare personnel must guide patients in managing health challenges and recommend further follow-up when necessary. Effective collaboration among DR-TB patients, hospital staff, primary healthcare and municipal entities is essential for facilitating a smoother transition to a healthy life and ensuring comprehensive support and care continuity.

### Strength and limitations

The study acknowledges limitations due to the lack of participation from some invited individuals, particularly those with language barriers and those, who declined after consulting with family members. Although the participants were comfortable with English or Norwegian during the interviews, many of them did not use their native languages, potentially restricting their expressions and nuances. Most of the participants underwent treatment several years ago and recall bias might occur. However, all participants spoke with commitment and were active during interviews. The interviewer made efforts to follow-up on questions and responses, when necessary, in order to avoid misunderstandings. To strengthen the results a participant with English as native language considered the results before final writing up. Despite challenges, the inclusion of 16 diverse participants is considered a strength, as it provided a rich variety of narratives that contributed to valuable insights into the research question. This variety ensured that the study captured a broad spectrum of perspectives on living with TB, enhancing its informational power and relevance. This study was carried out in a TB low-endemic country, and the findings cannot automatically be generalized to other settings.

## Conclusion

This study highlights the enduring impact of DR-TB on patients in Norway, emphasizing the importance of early diagnosis, the challenges with navigating healthcare systems and the necessity of psychosocial support for people during the diagnosis, treatment and recovery phases. Despite variations in their experiences, the participants faced serious psychosocial implications, including stigma and isolation, and reflected on missed early intervention opportunities. This study underscores the need for improved healthcare awareness of TB signs and symptoms, communication, and psychosocial support during and after completed treatment. We recommend further research conducted in TB low-endemic countries that evaluate the international and local recommendations on psychosocial support.

### Supplementary Information

Supplementary Material 1.

## Data Availability

The transcribed narratives of the participants are not publicly available due the participants’ confidentiality and anonymity. Fragments of the data material might be available on reasonable request to the corresponding author. The dataset is available five years after the end of the project in accordance with the REK’s ethical approval.
