# Supplementary material for: Beyond the diagnosis of drug-resistant Tuberculosis in Norway: patients’ experiences before, during and after treatment
Source: BMC Public Health. 2024 Jul 6;24:1801. doi: 10.1186/s12889-024-19342-8 (PMC11227233; doi:10.1186/s12889-024-19342-8)
Supplement: Supplementary file 1 — Supplementary Material 1. [file 12889_2024_19342_MOESM1_ESM.pdf]

**Beyond the Diagnosis: Exploring the Psychosocial Impact of Drug-Resistant Tuberculosis on Patients in Norway**

## Interview guide - Semi-structured

| Main area | Questions |
|---|---|
| Participant Classification | • Do you remember the year when you were treated for tuberculosis (TB)?<br>• How long did the treatment last?<br>• What is your country of birth?<br>• How many years did you live in Norway before the TB diagnosis? (Immigrants)<br>• How old are you?<br>• Do you work, study, or are you unemployed?<br>• What is your level of education?<br>• Do you live alone or with family?<br>• Did you live alone or with family during the TB treatment? |
| Experiences of undergoing TB disease and treatment | • How did you experience the period during diagnosis of TB?<br>• How did you experience the treatment period? |
| | Daily living at hospital and at home:<br>• If you were isolated at hospital, how did you experience this time?<br>• Describe your experiences of the disease in relation to daily activities at home, at school and at work.<br>• How did you experience the support of your family and friends during and after the disease?<br>• How did you experience support from school and job during and after the disease?<br>• How did you experience support from the health care during and after the disease?<br>• Describe how the disease affected your social network.<br>• How does the disease you underwent have an impact on what you do today?<br>• (Immigrants) Describe the impact of getting a TB diagnosis for you as an immigrant. |
| | Motivation and coping:<br>• How did you manage the treatment? |
| | Experiences of TB medications and Direct Observed Treatment (DOT):<br>• Describe your experiences with the TB medications.<br>• How did you manage the medications every day and DOT over a long period? |
| | Communications:<br>• Describe the communication with doctors, nurses and other health care personnel at hospital and in the municipality. |
| Consequences of TB diagnosis and treatment for the participants' health | • How would you describe your health during diagnosis and treatment?<br>• Describe actions that promoted your health.<br>• Describe measures that you missed.<br>• How did the disease and the treatment influence your health and well-being today? |

| | |
|---|---|
| | Focus: General-, physical-, social and mental health, and emotional aspect and pain |
| Summative questions | • It has been [X] years since you recovered from tuberculosis. Describe how you are today.<br>• What could you, your social network and the health care have done differently? |
